# Cessation of antithrombotic therapy before surgery: weighing thrombosis and bleeding risks

**DOI:** 10.1007/s12471-014-0580-6

**Published:** 2014-07-24

**Authors:** M. I. Meesters, C. Boer

**Affiliations:** Department of Anaesthesiology, Institute for Cardiovascular Research, VU University Medical Centre, De Boelelaan 1117, 1081 HV Amsterdam, the Netherlands

**Keywords:** Antithrombotic, Haemorrhage, Perioperative, Thrombosis, Surgery

Antithrombotic drugs are widely used for a variety of indications to prevent arterial and venous thrombosis. When antithrombotic drugs are discontinued in the days before a surgical procedure, the risk for perioperative bleeding may decrease, whereas the risk of thromboembolic events rises. The balancing of these risks remains an ongoing challenge for the perioperative healthcare professional.

The risk for perioperative bleeding when antithrombotic therapy is continued is determined by the type of surgery, patient comorbidities and the type of anticoagulation therapy [1]. Bleeding, and the consequent transfusion of blood products, are both independently associated with increased morbidity and mortality [2]. Moreover, the location of perioperative bleeding is an important contributing factor to outcome, as it may cause serious complications in vital organs, and be difficult to control.

Similarly, thromboembolic events may lead to complications such as pulmonary emboli, myocardial infarction or cerebral vascular events, all contributing to an unfavourable patient outcome. The risk for thrombotic events is based on the indication for anticoagulation therapy and patient characteristics, and increases in case of a surgical intervention [3].

Current guidelines assess the risk of thromboembolic events by treatment indication, and compare these with increased bleeding risks when anticoagulant therapy is continued. Yet, perioperative management of antithrombotic drugs remains controversial, as there is only limited evidence available for the right therapeutic decision. Moreover, studies investigating the risk of (dis)continuation of antithrombotic drugs frequently use different treatment protocols and definitions for bleeding and thromboembolic processes, resulting in ambiguous results.

The PRAGUE-14 registry gives more insight into the choice between the two evils in patients with cardiovascular disease [4]. Patients continuing anticoagulant drugs had twice as many bleeding events (13.3 %) compared with thrombotic events (7.6 %). However, these bleeding events led 10 times less often to death: 0.3 % from bleeding versus 3.0 % as a result of thrombotic events. This suggests that the increased risk for perioperative bleeding resulting from continuation of antithrombotic drugs seems to be manageable, whereas thromboembolic events have serious consequences.

Despite the interesting conclusions of the PRAGUE-14 study, the article fails to show important information that might be required to draw the right conclusions. First, while it is known that the impact of cessation of antithrombotic therapy partly depends on the preoperative health status of the patient, the authors refrained from presenting data on the specific interaction between preoperative comorbidities and the decision to stop antithrombotic therapy, in particular in light of a bridging protocol. For instance, the authors suggest in the discussion that previous percutaneous coronary intervention with coronary stent implantation is an independent risk factor for perioperative complications. However, it is unclear from their study whether they included patients with a coronary stent who stopped antithrombotic therapy before surgery, which may have a deleterious effect on outcome, leading to myocardial infarction and mortality. This information could be provided if the authors performed a specific analysis of the interaction between cessation of antithrombotic therapy and specific preoperative comorbidities. Moreover, it is unclear whether patients were subjected to a bridging protocol to prevent preoperative and postoperative thrombotic complications.

Secondly, different antithrombotic drugs exert a different impact on the risk for thrombosis or bleeding, and this risk is subsequently modulated by patient health status and the treatment regime. For instance, the recent randomised controlled POISE-2 trial, which is referred to in the discussion section of the PRAGUE-14 study, showed that the use of aspirin before surgery and throughout the early postsurgical period had no effect on mortality, but increased the risk of major bleeding with a hazard ratio of 1.23 [5]. Interestingly, there was no difference in mortality or major complications between patients who were already using aspirin before surgery, or started on aspirin for study purposes. However, a closer evaluation of the supplementary data of the POISE-2 trial shows that patients who were already on aspirin before surgery did not have an increased risk of bleeding, while patients who started on aspirin therapy before surgery showed a higher prevalence of major haemorrhage. These data suggest that it is complex to provide specific evidence for the benefits or disadvantages of antithrombotic drug cessation before surgery, and that the conclusions that are drawn from these studies should be considered in light of distinct circumstantial aspects.

In order to change the current guidelines and to provide practical guidance for the perioperative healthcare professional, more studies are required that specifically focus on the cessation or continuation of one antithrombotic drug in a more uniform surgical patient population. However, due to its reflection of daily practice, data obtained from large patient registries such as PRAGUE-14 may particularly be used to generate relevant hypotheses and study algorithms for future trials.
